# Weighted functional linear regression models for gene-based association analysis

**DOI:** 10.1371/journal.pone.0190486

**Published:** 2018-01-08

**Authors:** Nadezhda M. Belonogova, Gulnara R. Svishcheva, James F. Wilson, Harry Campbell, Tatiana I. Axenovich

**Affiliations:** 1 Institute of Cytology and Genetics, Siberian Branch of the Russian Academy of Sciences, Novosibirsk, Russia; 2 Vavilov Institute of General Genetics, the Russian Academy of Sciences, Moscow, Russia; 3 Centre for Global Health Research, Usher Institute for Population Health Sciences and Informatics, University of Edinburgh, Edinburgh, Scotland; 4 MRC Human Genetics Unit, Institute of Genetics and Molecular Medicine, University of Edinburgh, Western General Hospital, Edinburgh, Scotland; 5 Novosibirsk State University, Novosibirsk, Russia; National Institute of Environmental Health Sciences, UNITED STATES

## Abstract

Functional linear regression models are effectively used in gene-based association analysis of complex traits. These models combine information about individual genetic variants, taking into account their positions and reducing the influence of noise and/or observation errors. To increase the power of methods, where several differently informative components are combined, weights are introduced to give the advantage to more informative components. Allele-specific weights have been introduced to collapsing and kernel-based approaches to gene-based association analysis. Here we have for the first time introduced weights to functional linear regression models adapted for both independent and family samples. Using data simulated on the basis of GAW17 genotypes and weights defined by allele frequencies via the beta distribution, we demonstrated that type I errors correspond to declared values and that increasing the weights of causal variants allows the power of functional linear models to be increased. We applied the new method to real data on blood pressure from the ORCADES sample. Five of the six known genes with *P* < 0.1 in at least one analysis had lower *P* values with weighted models. Moreover, we found an association between diastolic blood pressure and the *VMP1* gene (*P* = 8.18×10^−6^), when we used a weighted functional model. For this gene, the unweighted functional and weighted kernel-based models had *P* = 0.004 and 0.006, respectively. The new method has been implemented in the program package FREGAT, which is freely available at https://cran.r-project.org/web/packages/FREGAT/index.html.

## Introduction

Rapid progress in next-generation whole-exome and whole-genome sequencing technologies provides new opportunities for detection of rare genetic variants that control complex traits. However, statistical methods using single-variant association tests that are commonly adopted in genome-wide association studies are generally underpowered for rare variants. The statistical power of association analysis increases when the genetic variants in a genomic region are tested all at once, not individually [1, 2].

Several approaches have been proposed for region-based association analysis of rare variants. These include burden tests based on collapsing rare variants within a region [2–6], the kernel machine technique based on variance component analysis [7–11], and functional data analysis (FDA) using a continuous functional description of sets of discrete real data [12–16]. Each of these approaches has its own advantages and disadvantages. The collapsing-based methods are the fastest and simplest. They can be very powerful when the majority of variants are causal and their effects are unidirectional. The power of association analysis decreases if these assumptions do not hold [17]. In contrast to collapsing-based methods, the kernel-based methods are more resistant to the opposite direction of causal variant effects and the limited proportion of causal variants [11, 18, 19]. However, they are more complex than collapsing-based methods computationally.

Methods using the FDA approach have additional advantages. Their main rationale is reduction of the influence of noise and/or observation errors [20]. Moreover, they consider not only the genotypes of multiple genetic variants within a particular genomic region, but also the physical locations of these variants, that is, the order of these variants and the distances between them [12, 13]. These methods are expected to be particularly effective for regions with a large number of genetic variants because they reduce the number of estimated parameters [20].

The key of all gene-based methods is to combine information about the association between the trait and genotypes of every genetic variant to calculate a single value of the statistical test for the entire region. This pooling can be achieved by merging genotypes for collapsing-based methods [21], combining score-tests for the kernel-based methods [22] or constructing continuous smoothing functions for FDA-based methods [20].

For methods where information of different genetic variants about a tested hypothesis is summarized, the variant weights can be introduced to the model. Good choices of weights can improve power. The weights are prespecified using any kind of data (for example, genotypes, covariates or external biological information) that is estimated without using the outcome and reflects the relative contribution of each variant to the test statistics [11]. Weights allow introduction of any a priori information on which variants are more likely to be causal. This can yield improved power.

Weights have been introduced to the models assuming random genotype effects: collapsing-based and kernel-based methods. However, none of the models assuming fixed genotype effects use weights.

In this paper, we introduce weights to a functional linear regression model of fixed genotype effects described for testing an association using both independent and structured samples. We estimate the statistical properties of our new method using Genetic Analysis Workshop 17 mini-exome independent and family data [23] and a wide range of simulation scenarios. Additionally, we apply the new method to real data on blood pressure in the Orkney Complex Disease Study (ORCADES) sample [24].

## Weighted functional linear regression model

Consider a genomic region containing *m* genetic variants with known physical locations *t*_*i*_ (*i* = 1, …, *m*). Let the genetic variants be ordered as *t*_1_<…<*t*_*m*_ and scaled from [*t*_1_, *t*_*m*_] to [0, 1].

For a sample of *n* individuals, let *y* denote an (*n*×1) vector of known trait values, *X* denote an (*n*×(1+*c*)) matrix, in which the first column is a vector of units and the other columns are *c* covariates, and *G* denote an (*n*×*m*) matrix of genotypes of *m* variants. Here, *G*_*ij*_ is equal to the number of minor alleles of the *i-*th individual for the *j*-th variant with the location *t*_*j*_.

The traditional linear regression model of multiple additive effects for an arbitrarily structured sample of *n* individuals is expressed as:

y=Xα+Gβ+h+ε. (1)

Here *α* is a fixed ((1+*c*)×1) vector of regression coefficients, whose first element measures the intercept and the others measure the effects of *c* covariates; *β* is an (*m*×1) vector of regression coefficients describing the fixed effects of *m* genetic variants; *h* is an (*n*×1) random vector of polygenic effects distributed as *N*(0; *σ*^2^_*g*_*R*), and *ε* is an (*n*×1) random vector of errors distributed as *N*(0; *σ*^2^_*e*_*I*), where *σ*^2^_*g*_ and *σ*^2^_*e*_ are the respective components of the total variance *σ*^2^ = *σ*^2^_*g*_ + *σ*^2^_*e*_ of the trait. Here *R* and *I* are an (*n*×*n*) relationship and identity matrices, respectively. Model (1) assumes that the phenotypes *y* follow a multivariate normal distribution with a mean vector *E*(*y*) = *Xα* + *Gβ* and a covariance matrix *Ω* = *σ*^2^_*g*_*R* + *σ*^2^_*e*_*I*. If the sample consists of unrelated individuals, then *R = I* and Ω = *σ*^2^*I*.

In the framework of the FDA approach, discrete genotypic values of ordered variants (for each individual) and effects of the variants are interpreted as continuous data [12]. In this case, a functional linear regression model (FLM) is defined as

$$y=X\alpha +\int_{0}^{1}\tilde{G}\left(t\right)\tilde{\beta }\left(t\right)dt+h+\varepsilon .$$ (2)

Here $\tilde{G}(t)={\left({\tilde{G}}_{1}(t),\ldots ,{\tilde{G}}_{n}(t)\right)}^{T}$ denotes an (*n*×1) unknown vector of genetic variant functions (GVFs), and $\tilde{\beta }(t)$ denotes an unknown beta-smoothing function (BSF) of *t* in [0,1].

By applying FDA, GVFs and BSF can be described by sets of *K*_*G*_ and *K*_*β*_ basis functions, respectively. According to [14], $\tilde{G}(t)$ and $\tilde{\beta }(t)$ are estimated as

$$\tilde{G}(t)=G\Phi {\left(\Phi^{T}\Phi \right)}^{-1}\phi (t)$$

and

$$\tilde{\beta }(t)=\psi^{T}(t)\beta_{F},$$

where *ϕ*(*t*) = (*ϕ*_1_(*t*),…,*ϕ*_*KG*_(*t*))^*T*^ is a (*K*_*G*_×1) vector of basis functions that are used to smooth the genotypes; *Φ* is an (*m×K*_*G*_) matrix with an element *Φ*_*ij*_ = *ϕ*_*j*_(*t*_*i*_); *ψ*(*t*) = (*ψ*_1_(*t*),…,*ψ*_*Kβ*_(*t*))^*T*^ is a (*K*_*β*_×1) vector of basis functions that are used to smooth the genetic effects; and, finally, $\beta_{F}={\left(\beta_{F_{1}},\ldots ,\beta_{F_{K_{\beta }}}\right)}^{T}$ is a (*K*_*β*_×1) vector of model regression coefficients.

Substituting the expressions for $\tilde{G}(t)$ and $\tilde{\beta }(t)$ to Eq (2) yields

$$y=X\alpha +GW\beta_{F}+h+\varepsilon ,$$ (3)

where

$$W=\Phi {\left(\Phi^{T}\Phi \right)}^{-1}\int_{0}^{1}\phi \left(t\right)\psi^{T}\left(t\right)dt.$$

The (*m*×*K*_*β*_) smoother-matrix *W* is formed from two sets of basis functions, *ϕ*(*t*) and *ψ*(*t*), intended for smoothing genotypes and their effects, respectively. It depends on the type and number of the predefined basis functions, as well as on the positions of genetic variants in the region. In fact, the matrix *W* converts the (*n*×*m*) design matrix, where each row is a set of *m* individual’s real genotypes, into a new (*n*×*K*_*β*_) design matrix where each row is a set of *K*_*β*_ linear combinations of *m* real genotypes. So, models (1) and (3) differ by region-specific genetic components: *Gβ* versus *GWβ*_*F*_. Moreover, the parameters associated with genotype effects appear as vector *β*_*F*_ of size (*K*_*β*_×1) in model (3) and as vector *β* of size (*m*×1) in model (1).

Usually, identical sets of basis functions (being equal in type and number) are used for GVFs and BSF. In this case, the model with both genotypes and their effect smoothed becomes equivalent to that without genotypes smoothing (the beta-smooth only model) [15]. However, different types and/or numbers of basis functions may be used for GVFs and BSF.

Model (3) assumes that the phenotypes *y* follow a multivariate normal distribution with the mean vector *E*(*y*) = *Xα* + *GWβ*_*F*_ and the covariance matrix *Ω* = *σ*^2^_*g*_*R* + *σ*^2^_*e*_*I*. In this case, two hypotheses are compared, H0: *β*_*F*_ = 0 versus H1: *β*_*F*_ ≠ 0. The number of parameters of interest is *K*_*β*_ in model (3) and *m* in model (1). See more details about association analysis using FLM in [12, 14].

We modified model (3) by introducing to it weights preset for every genetic variant:

$$y=X\alpha +G\Theta W\beta_{F}+h+\varepsilon .$$ (4)

Here *Θ* is an (*m*×*m*) diagonal matrix of weights for *m* genetic variants. The new smoothing matrix *ΘW* in model (4) is constructed not only using the values of the given basis functions at the positions of the genetic variants, but also using the weights predefined for every genetic variant. The introduction of the diagonal matrix of weights modifies the (*n*×*K*_*β*_) design matrix. Before the weighing procedure, each element of the design matrix (*GW*) represents some linear combination of *m* real genotypes. In the new design matrix (*GΘW*), the influence of each genetic variant in this combination is controlled by weight prespecified for this variant. For the variants with higher weights, their impact in the design matrix is higher than that in model (3) as well as for the variants with lower weights, their impact in the design matrix is lower than that in model (3).

The stronger the differences between weights of causal and non-causal variants, the higher the power of an association test. A simplest a priori supposition about what variants are causal is that deleterious mutations are expected to be rare. In this case, the weights are defined by the minor-allele frequency (MAF), for example, as the beta distribution density function √*w*_*j*_ = Beta(MAF_*j*_; *a*_1_, *a*_2_) with the prespecified parameters *a*_1_ and *a*_2_ evaluated at MAF for the *j*-th variant [11].

## Statistical properties of the method

We compared two standard beta-smooth only models: 15 B-spline or 25 Fourier basis functions. Such numbers of B-spline and Fourier basis functions have been recommended by Fan et al. [12] and tested in our previous study [14] (see [14] for discussion on the optimal choice of the number of the basis functions (*K*)).

We used genotypes of Genetic Analysis Workshop 17 (GAW17 [23]) family-based and population samples, each consisting of 697 individuals. The trait was modelled as random realization from the multivariate normal distribution *N*(*Gβ*, *h*^2^*R* + (1 –*h*^2^)*I*), where *G* is a matrix of genotypes for variants selected to be causal, *β* is a vector of additive effect sizes of genetic variants, *R* and *I* are the relationship and identity matrices (*R = I* for the population sample), *h*^2^ is heritability (we set *h*^2^ = 0.29, as in the GAW17 quantitative trait Q2).

To estimate the type I error, we simulated the trait without effects of genetic variants (*β* = 0). We analyzed gene regions with > 25 polymorphic exome genetic variants of the GAW17 data sets to avoid overparametrization under chosen *K*_*β*_ = 25 (see [14] for details). In the population sample, we analyzed 215 gene regions (9,909 genetic variants in total) and simulated 1×10^5^ replicates under the null hypothesis to obtain 2.15×10^7^ regional *P* values. In family sample, we analyzed 60 genes (2,598 genetic variants in total) and simulated 5×10^5^ replicates to obtain 3×10^7^ regional *P* values. The type I error was estimated as the proportion of *P* values that are less than alpha, with alpha ranging from 0.05 to 2.5×10^−6^.

Table 1 shows type I errors obtained as the proportion of the simulations of the null hypothesis with *P* ≤ α. The type I errors are very close to the declared levels.

**Table 1:** Type I error rates of weighted FLM tests*.

Alpha	Population sample		Family sample	
	B-spline	Fourier	B-spline	Fourier
0.05	0.049698	0.040754	0.04952	0.044309
0.01	0.009905	0.007961	0.009793	0.008516
0.001	0.000978	0.000793	0.00094	0.000797
10^−4^	8.79×10^−5^	7.77×10^−5^	8.93×10^−5^	7.47×10^−5^
10^−5^	8.37×10^−6^	7.44×10^−6^	8.57×10^−6^	6.80×10^−6^
2.5 × 10^−6^	1.40×10^−6^	1.40×10^−6^	1.77×10^−6^	1.40×10^−6^

*The standard weighted function defined by the beta distribution with *a*_1_ = 1 and *a*_2_ = 25 was used.

For power estimation, we selected gene regions that contained ≥ 30 polymorphic genetic variants and ≥ 10 rare variants with MAFs ≤ 0.03 (41 and 146 gene regions in the family and population samples, respectively). In each replicate, we randomly selected one of these regions for simulation of the region-specific genetic component of the trait (*Gβ* in the above formula). The following scenarios were considered: 1) the proportion of causal variants in the regions is 0.05, 0.1, or 0.2; 2) the proportion of effects that have the same direction is 0.5, 0.8, or 1; 3) either all genetic variants or only rare variants with MAFs ≤ 0.03 used to select causal variants; 4) for each causal variant *j*, the effect size was simulated as (*i*) |*β*_*j*_| = log(*s*)|log_10_(MAF_*j*_)|/2 similar to [12], with *s* being equal to 2, 3, 5, or 7 (larger *β* for lower MAF, but still a lower proportion of variance explained by rare variants) or as (*ii*) $|\beta_{j}|=\sqrt{s/2{MAF}_{j}(1-{MAF}_{j})}$, with *s* being equal to 0.01, 0.02, 0.03, 0.05, or 0.1 (the same proportion of variance explained by each causal variant).

We compared the powers of the new method and the unweighted FLM test. The latter has been well studied and shown to be more powerful than collapsing and kernel-based methods for many simulated scenarios [12–14].

We analyzed the association between the quantitative traits and the genotypes of genetic variants in the region using *F*-statistics for testing fixed effects in the mixed model. Under each scenario, the power was estimated as the proportion of *P* values that were less than 2.5×10^−6^ in 2000 replicates.

**Fig 1:**
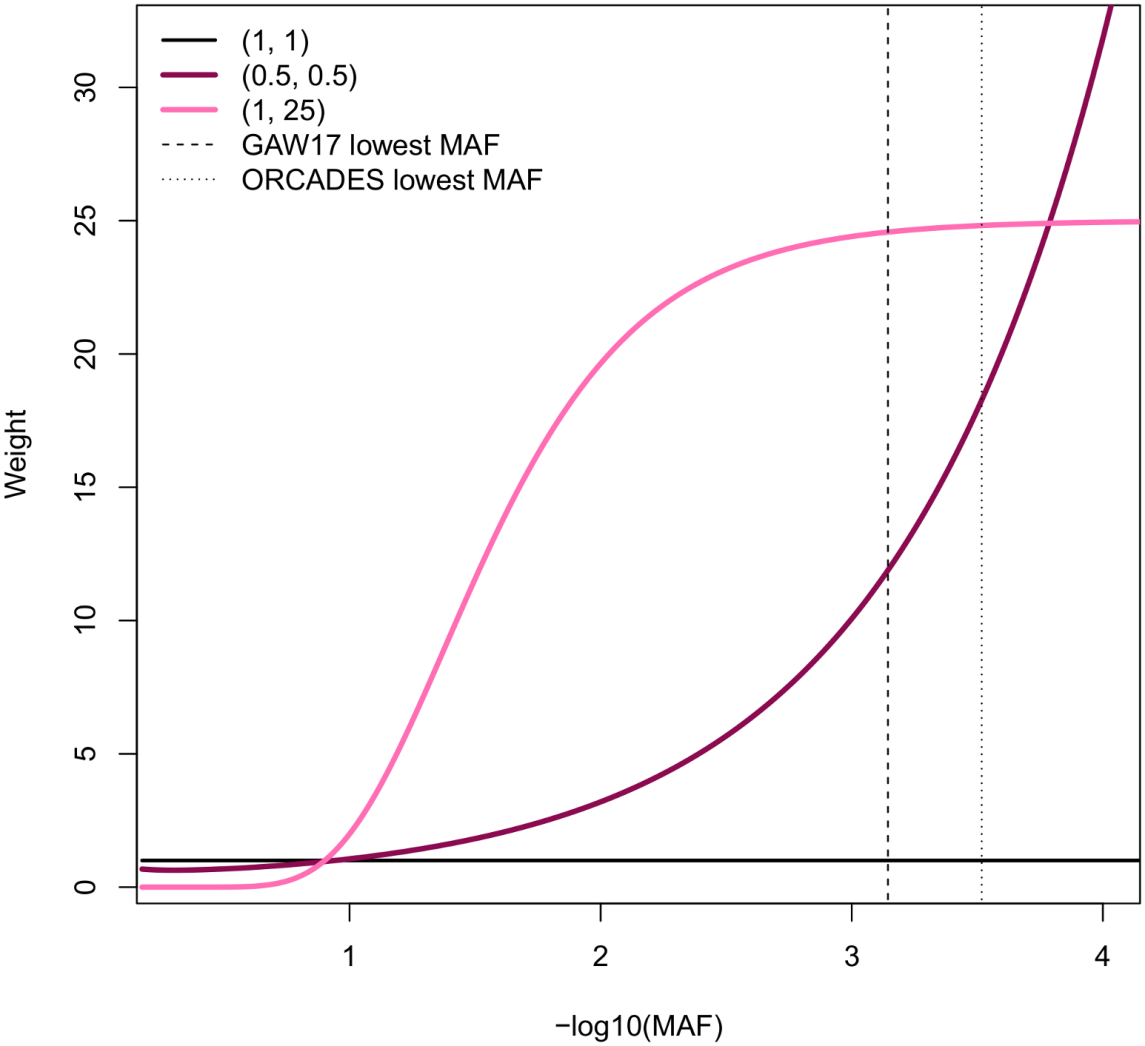
Weights calculated as Beta(MAF; *a*_1_, *a*_2_) for three weighting modes. Numbers in parentheses are the values of the beta function parameters *a*_1_ and *a*_2_.

All the power estimates were made for the weighted models with two standard weighting parameter sets (*a*_1_, *a*_2_): 1) *a*_1_ = *a*_2_ = 0.5 and 2) *a*_1_ = 1; *a*_2_ = 25. To varying degrees, these functions give more weights to rare variants (Fig 1). The beta function parameters *a*_1_ = *a*_2_ = 1 were used to represent a standard unweighted FLM. Each weighting function was tested for two standard beta-smooth only FLM: 15 B-spline and 25 Fourier basis functions. Analysis was performed using the FREGAT package [25].

**Fig 2:**
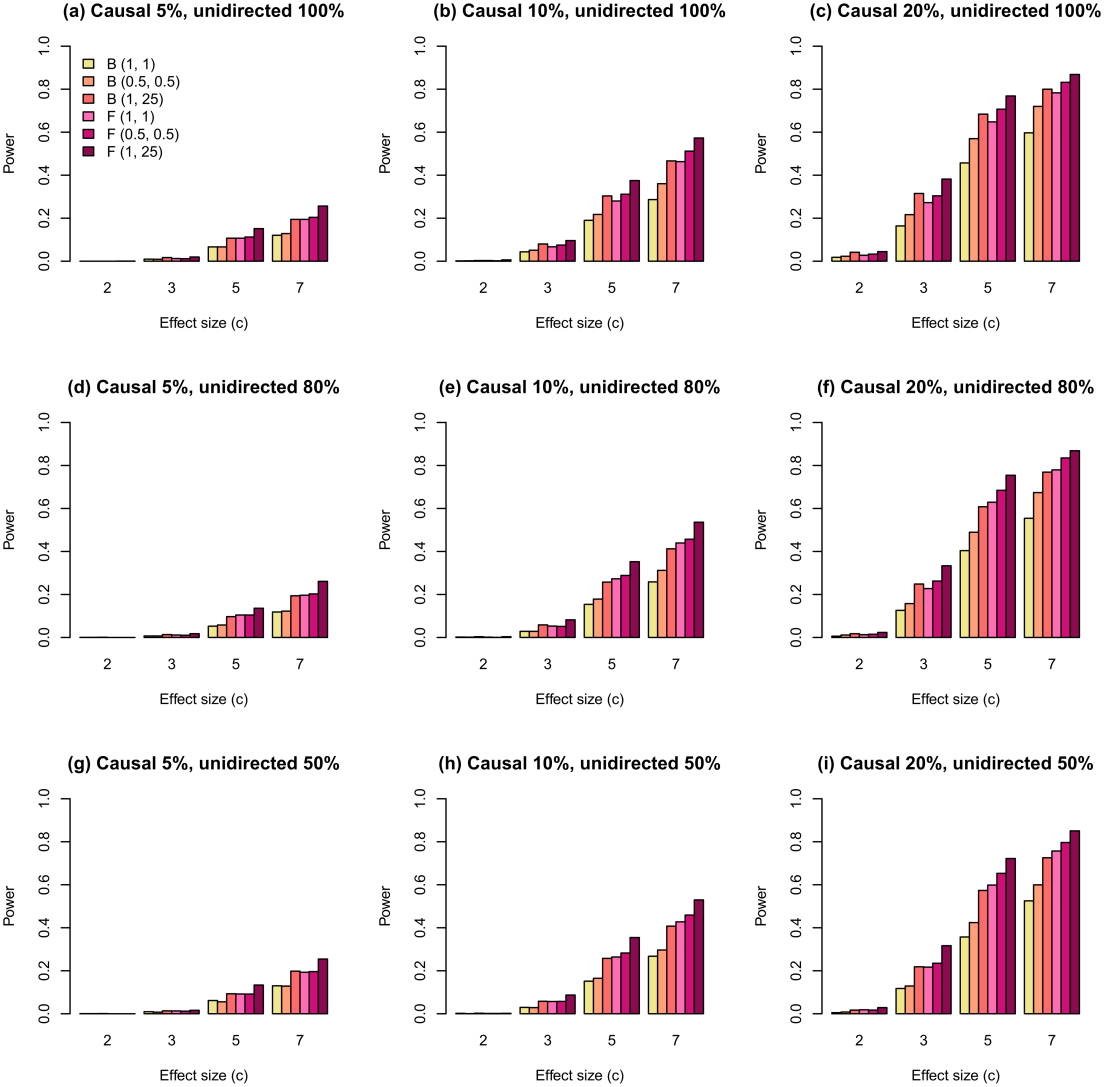
The statistical power of regional association analysis with weighted FLM on the familial data with effect modeled as |*β*_*j*_| = log(*s*)|log_10_(MAF_*j*_)|/2 and all causal variants having MAFs ≤ 0.03. Proportion of causal variants is the proportion of all rare variants (MAF ≤ 0.03) within the region (all rare variants = 100%). B—B-spline basis functions; F—Fourier basis functions; (1, 1)—the unweighted model; (0.5, 0.5)—the weighted model with *a*_1_ = *a*_2_ = 0.5; (1, 25)—the weighted model with *a*_1_ = 1 and *a*_2_ = 25.

Fig 2 and S1 Fig illustrate the powers of the tested models under different scenarios in family and population data, respectively. All causal variants had MAFs ≤ 0.03 and the effect size of the *j-*th variant was modeled as |*β*_*j*_| = log(*s*)|log_10_(MAF_*j*_)|/2. Increasing the weights of rare (causal) variants allows increasing the power of functional linear models. For the test using the weighting function with the parameters *a*_1_ = 1 and *a*_2_ = 25, the power is higher than the powers of the unweighted test for all the scenarios. For family data, the test using the weighting function with *a*_1_ = *a*_2_ = 0.5 has the power higher than does the unweighted model, but lower than does the weighted model with *a*_1_ = 1 and *a*_2_ = 25. For population data, the effect of weighting is less than that for the family sample, and weighting with the parameters *a*_1_ = *a*_2_ = 0.5 seems to have advantage over the unweighted model only for large effect sizes. This pattern is consistent for both types of basis functions.

**Fig 3:**
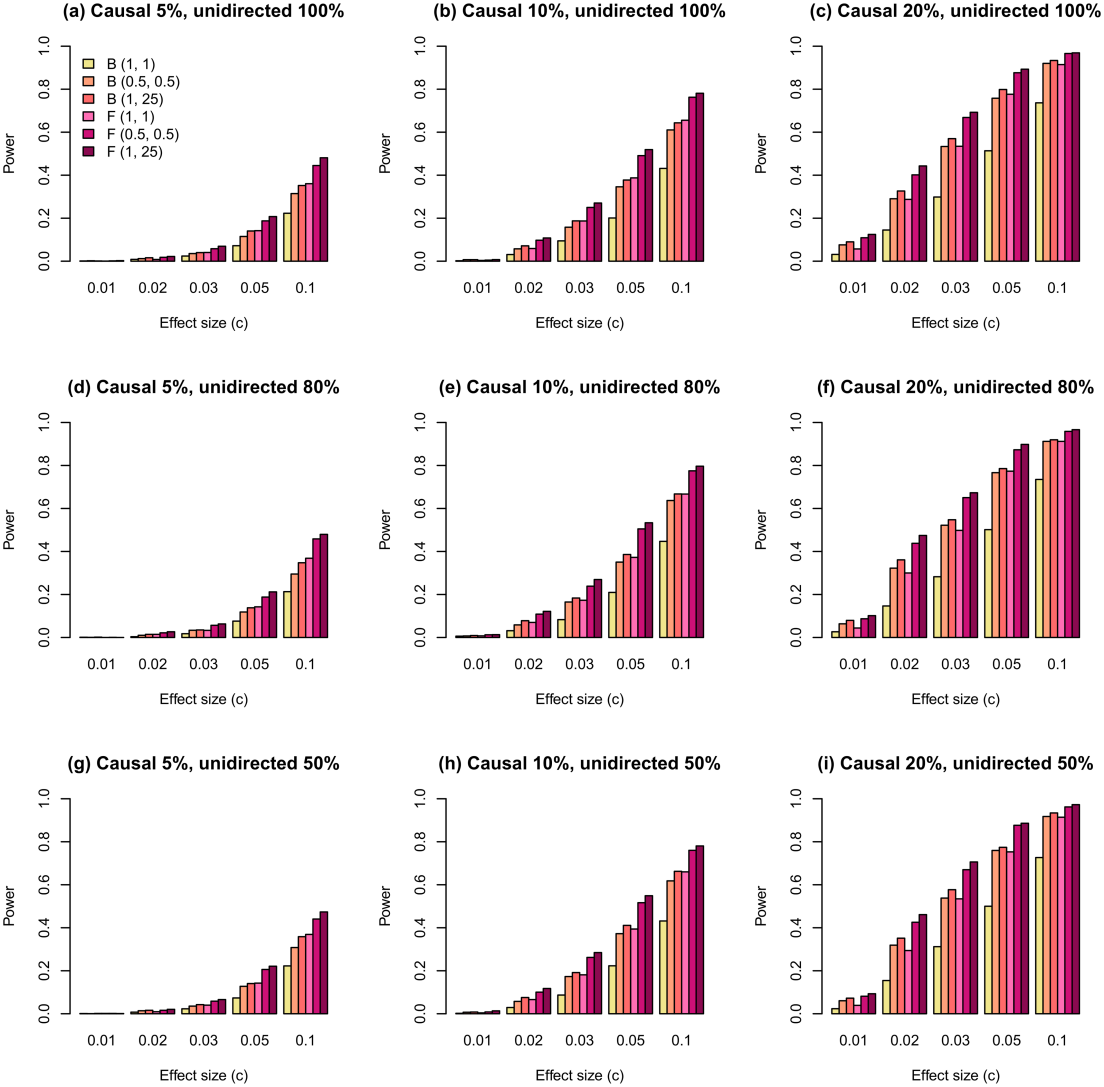
The statistical power of regional association analysis with weighted FLM on the familial data with effect modeled as $|\beta_{j}|=\sqrt{s/2{\text{MAF}}_{j}(1-{\text{MAF}}_{j})}$ and all causal variants having MAFs ≤ 0.03. Other model parameters and the notations are the same as in Fig 2.

The same effect of weighting was observed for scenarios, where the effect size was simulated as $|\beta_{j}|=\sqrt{s/2{MAF}_{j}(1-{MAF}_{j})}$ (Fig 3). In this case, the difference between the powers of weighted and unweighted models was higher than that for previously described scenarios (compare Figs 2 and 3).

We estimated the effect of weighting on the scenarios where causal variants were selected from both rare and common variants. The weighted models demonstrate an increased power only for the scenarios where the effect size was simulated as $|\beta_{j}|=\sqrt{s/2{MAF}_{j}(1-{MAF}_{j})}$ (S2 Fig). For the scenarios where the effect size was simulated as |*β*_*j*_| = log(*s*)|log_10_(MAF_*j*_)|/2, we did not observe an increase in power (S3 Fig). For all these scenarios, the weighting function with *a*_1_ = *a*_2_ = 0.5 demonstrates a higher power than does that with *a*_1_ = 1, *a*_2_ = 25. Additionally, we used a filtering technique when common variants (MAF > 0.03) were excluded from analysis. S2 and S3 Figs show that in this case the filtering technique is less effective than the weighting procedure.

As can be seen, for most scenarios, the models using Fourier basis show a higher power than the models using B-spline basis. This can be in part explained by a different number of basis functions: 15 for B spline and 25 for Fourier bases. However, we compared the power of these two models using the same numbers of basis functions and demonstrated that the models using Fourier basis have a higher power for both 15 and 25 basis functions (S4 Fig). Therefore, the different number of B-spline and Fourier basis functions cannot fully explain the difference in power. The decreased power of models using B-spline basis might be due to a uniform distribution of knots used in our study (see [14] for details). It is known that the power can be increased by the optimal choice of knots [20].

## Real data analysis

We analyzed blood pressure traits measured in the ORCADES sample [24, 26]. The study has ethical approval from NHS Orkney. All participants provided written, informed consent prior to participation. 1647 people were measured for SBP and 1645 for DBP. We considered 14,640 genes containing > 25 (mean 182.5) genetic variants suitable for the chosen FDA-based models. We used the same functional models as were applied to the simulated data: 15 B-spline basis functions or 25 Fourier functions; an unweighted model or models with the weighting function parameters *a*_1_ = 1; *a*_2_ = 25 or *a*_1_ = *a*_2_ = 0.5. No loci reached a common sense Bonferroni-corrected significance level in an exome-wide screen of the BP traits. Two loci reached *P* < 10^−5^ for DBP: *P* = 8.2×10^−6^ for the *VMP1* gene (encoding vacuole membrane protein 1) with the B15 model and the weighting function parameters *a*_1_ = 1; *a*_2_ = 25, and *P* = 9.1×10^−6^ for the *MC1R* gene with the unweighted F25 model. The unweighted functional and weighted kernel-based models had *P* = 0.004 and 0.006, respectively, for the *VMP1* gene. *MC1R* has already been found to be associated with heart failure (https://www.ncbi.nlm.nih.gov/projects/SNP/GaPBrowser_prod/callGaPBrowser2.cgi?snp=885479&aid=2884). For *VMP1*, an association with lipoprotein-associated phospholipase A2 activity (a marker of increased cardiovascular risk) has been shown [27].

**Fig 4:**
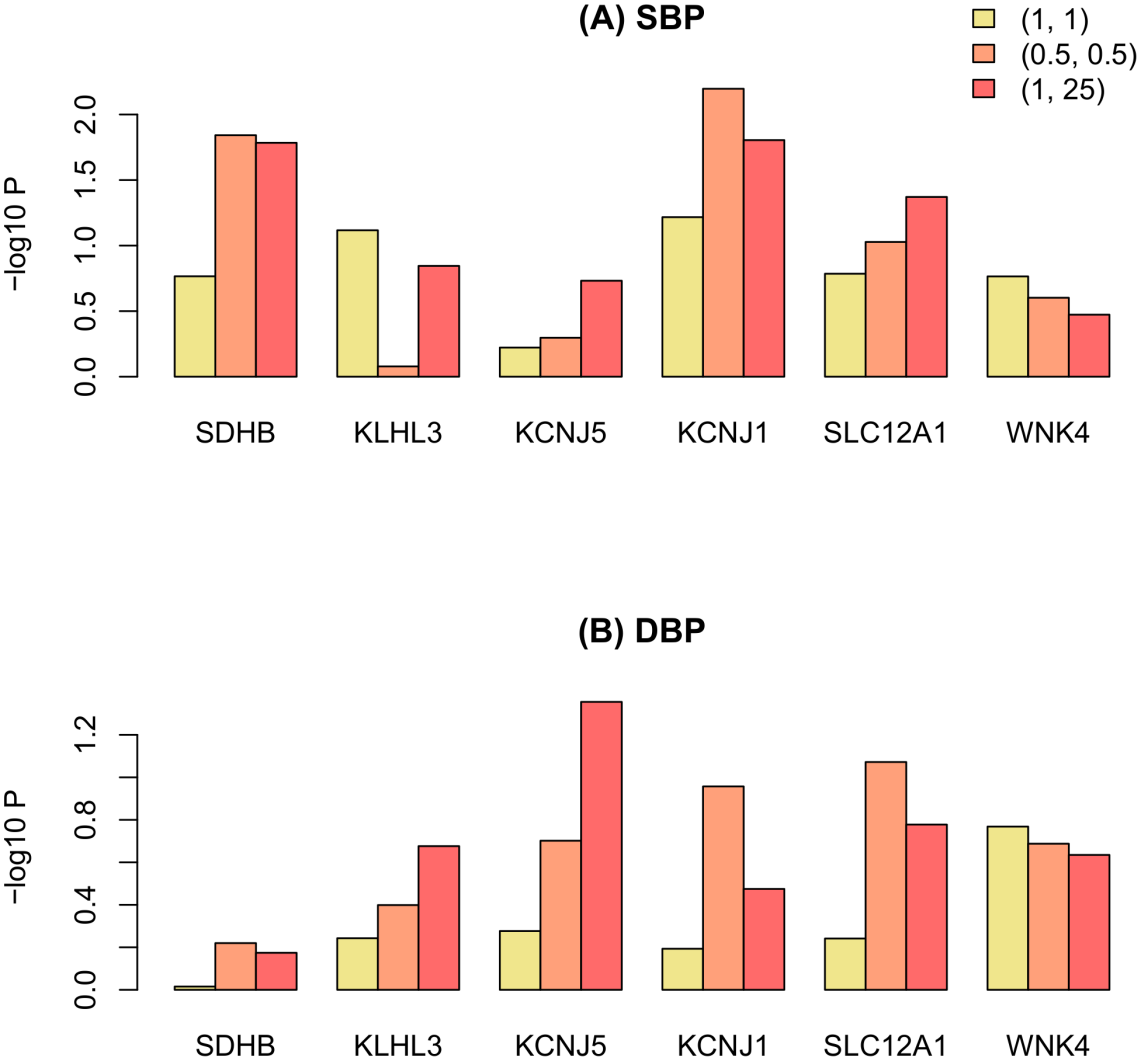
The results of regional association analysis of the known Mendelian BP genes having *P* < 0.1 in at least one analysis. The differently weighted FLM based on the Fourier basis functions was used.

For positive control, we looked at 28 known Mendelian BP genes [28]. 25 of these genes were available within the sample; 6 of them had *P* < 0.1 in at least one analysis. Fig 4 and S5 Fig show the results of differently weighted functional models for these genes. The models demonstrate gene-specific patterns. Five of these six genes (*SDHB*, *KCNJ5* and *SLC12A1* always, *KCNJ1* and *KLHL3* in most cases) had lower *P* values with weighted than unweighted models. The unweighted model was always better for *WNK4*, although there was no large difference between three models: unweighted, weighted with *a*_1_ = *a*_2_ = 0.5 and weighted with *a*_1_ = 1; *a*_2_ = 25. The Fourier and B-spline models showed similar behavior—as did the models with two types of weights.

## Discussion

We proposed a new weighted functional linear model for gene-based association analysis and demonstrated that the power of existing methods can be increased by introducing weights to functional linear models.

Our new model is the first weighted model with fixed genotype effects for region-based association analysis. Although weighting of predictors into the complete multiple linear regression model is meaningless, we showed how weights can be introduced into reduced models such as FLM. We propose that our weighting procedure can be generalized to other models of the same class, e.g. to principal component analysis based models. To date, no attempt has been made to increase their power with the help of weights assigned to different genetic variants in a way similar to what was successfully done for the models using collapsing and variance component approaches [4, 21, 22]. We show that weights can be introduced to functional linear regression models. Our findings suggest that this weighting can be beneficial and allows identification of additional loci that are not found with unweighted FLM or kernel-based methods.

The weights are defined as allele-specific coefficients that control the relative importance of each variant to test the association. Only some of the variants in the region are causal, and the rationale for introducing weights is to increase the impact of exactly these variants in the test statistics.

In addition to the common disease—common variants hypothesis, the common disease—rare variants hypothesis has been proposed to explain missing heritability [29]. The latter hypothesis assumes that complex traits are caused collectively by multiple rare variants with moderate to high penetrance. Under this hypothesis, it was proposed that rare genetic variants are more likely to be causal. Therefore, without a priori information about causality of the variants, the weights can be defined on the basis of allele frequencies, for example, via the beta distribution density function √*w*_*j*_ = Beta(MAF_*j*_; *a*_1_, *a*_2_) with the prespecified parameters *a*_1_ and *a*_2_ evaluated at the MAF for the *j-*th variant. The beta density is flexible and can accommodate a broad range of scenarios. By setting 0 < *a*_1_ ≤ 1 and *a*_2_ ≥ 1, the weight of each rarer variant can be increased and the weight of each common variant, decreased. Normally the *a*_1_ = 1 and *a*_2_ = 25 values are used in the kernel-based methods, because in this case the weight of each rare variant is increased, while for variants with MAF 1%–5% still put decent nonzero weights [11]. We have seen that the model with the weighting function parameters *a*_1_ = 1 and *a*_2_ = 25 had the highest power under many simulation scenarios, but this benefit was not obvious on real data. This could be explained by the difference in sample sizes and, therefore, MAFs. As it can be seen in Fig 1, models with the parameters *a*_1_ = 1 and *a*_2_ = 25 differentiate well between MAFs from 0.1 to 0.001, but assign almost equal weights to all MAFs < 0.001. On the contrary, model with *a*_1_ = *a*_2_ = 0.5 assigns increasingly higher weights to lower MAFs.

The effectiveness of the weighted FLM also depends on the effect sizes of common variants. We demonstrated that weighting by MAFs increased the power only in those scenarios where the difference between effect sizes (*β*) of rare and common variants was large. When the effect size of common variants is not small, weighted models can be ineffective. However, in this case genetic variants can easily be identified by single point association analysis. Regional association analysis has been specially proposed to identify rare genetic variants.

Recently, a filtering technique has become popular. With this technique, common variants in the study region are excluded from consideration [30]. Using the set of scenarios where the traits were simulated on both rare and common variants, we showed that weighting is preferable over filtering as it does not totally reject the information on the variants with lower weights (S2 and S3 Figs). Wu and the colleagues [11] have drawn the same conclusions. Filtering can be viewed as an extreme case of weighting. For example, a logistic weighting function with the parameter values 0.07 and 150 has recently been proposed [31]. In fact, it filters variants with MAF < 0.1 at these parameter values.

Good choices of weights can improve power. However, different weights may be optimal for different regions, as we have demonstrated with real data. The same behavior can be observed for weighted SKAT models [32, 33]. Therefore, we cannot expect that the weights would increase the test statistics for all causal regions. The good choice of weights problem is a particular case of the good choice of test problem. Many association tests have been proposed for gene-based analysis, but the choice of the most powerful test is uncertain because usually we have not enough information on the underlying genetic model. In our study, FDA-based models have relatively low *P* values for *VMP1* and *MC1R*, while the SKAT *P* values for these genes are > 10^-4^ in all three weighted models. On the other hand, unweighted SKAT detected an association of the *CRHR2* gene with SBP (*P* = 3.8×10^−6^), while all FDA-based models showed *P* values from 0.05 to 2.5×10^−5^ for this gene. FDA-based and kernel-based methods gather different relevant information. They model fixed and random effects, respectively, and often identify different loci. To put together the advantages of different tests, new methods for their combining have been proposed [32, 34]. Our weighted FDA-based model extends the list of gene-based association tests, which can be used for such testing.

The proposed weighting via MAFs is the simplest and does not require any additional research activity. Even so, it still appears to gain power when its assumption holds. Good a priori knowledge about what genetic variants are more likely to be causal would allow for even better efficiency. If a priori information is available, for example, some variants are predicted as functional, damaging or loss-of-function via Polyphen-2 [35] or other bioinformatic predictors, weights can be selected to increase the impact of likely functional variants [36, 37].

## Supporting information

S1 FigThe statistical power of regional association analysis with weighted FLM on population data with effect size modeled as $|\beta_{j}|=\sqrt{s/2{MAF}_{j}(1-{MAF}_{j})}$.Other model parameters and notations are as in Fig 2.(TIF)

S2 FigThe statistical power of regional association analysis with weighted FLM using familial data with effect size modeled as $|\beta_{j}|=\sqrt{s/2{MAF}_{j}(1-{MAF}_{j})}$ using both rare and common variants.Proportion of causal variants is the proportion of all variants within the region (all variants = 100%). Other model parameters and notations are as in Fig 2.(TIF)

S3 Fig The statistical power of regional association analysis with weighted FLM on familial data with effect modeled as |*β*_*j*_| = log(*s*)|log_10_(MAF_*j*_)|/2 using both rare and common variants. Proportion of causal variants is the proportion of all variants within the region (all variants = 100%). Other model parameters and notations are as in Fig 2.
(TIF)

S4 Fig The statistical power of regional association analysis for different numbers of basis functions (*K*_*β*_). **Unweighted FLM was used on familial data**. B: B-spline basis functions; F: Fourier basis functions. The effect size for the *j-*th variant was modeled as |*β*_*j*_| = log(*s*)|log_10_(MAF_*j*_)|/2 using rare variants. Other model parameters and notations are as in Fig 2.
(TIF)

S5 Fig The results of regional association analysis of the known Mendelian BP genes having *P* < 0.1 in at least one analysis. The differently weighted FLM based on the B-spline basis functions was used. The notations of the models are the same as in Fig 2.
(TIF)
