# Supplementary figures and images for: Weighted functional linear regression models for gene-based association analysis
Source: PLoS One. 2018 Jan 8;13(1):e0190486. doi: 10.1371/journal.pone.0190486 (PMC5757938; doi:10.1371/journal.pone.0190486)

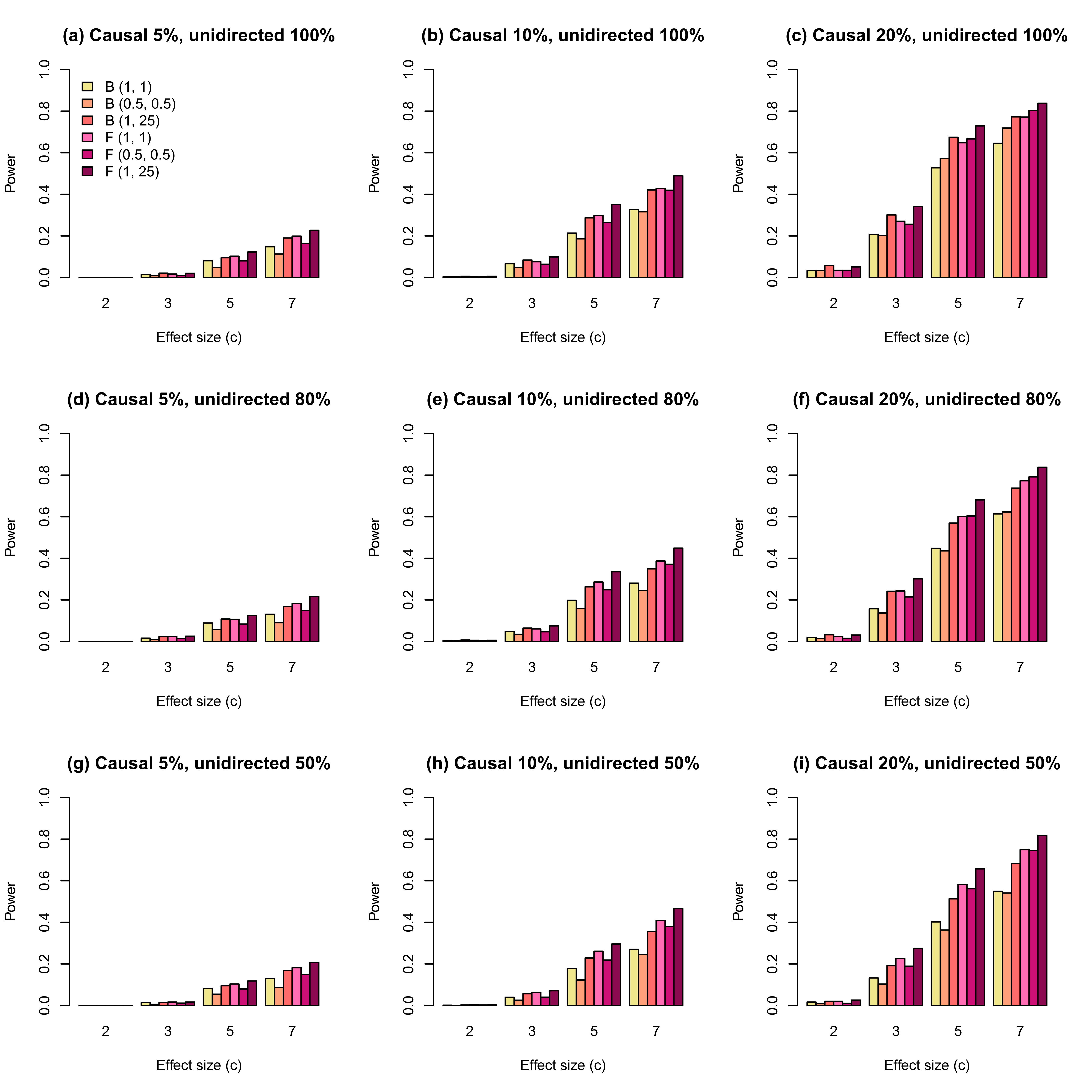

Supplement: S1 Fig — Other model parameters and notations are as in Fig 2. (TIF) [file pone.0190486.s001.tif]

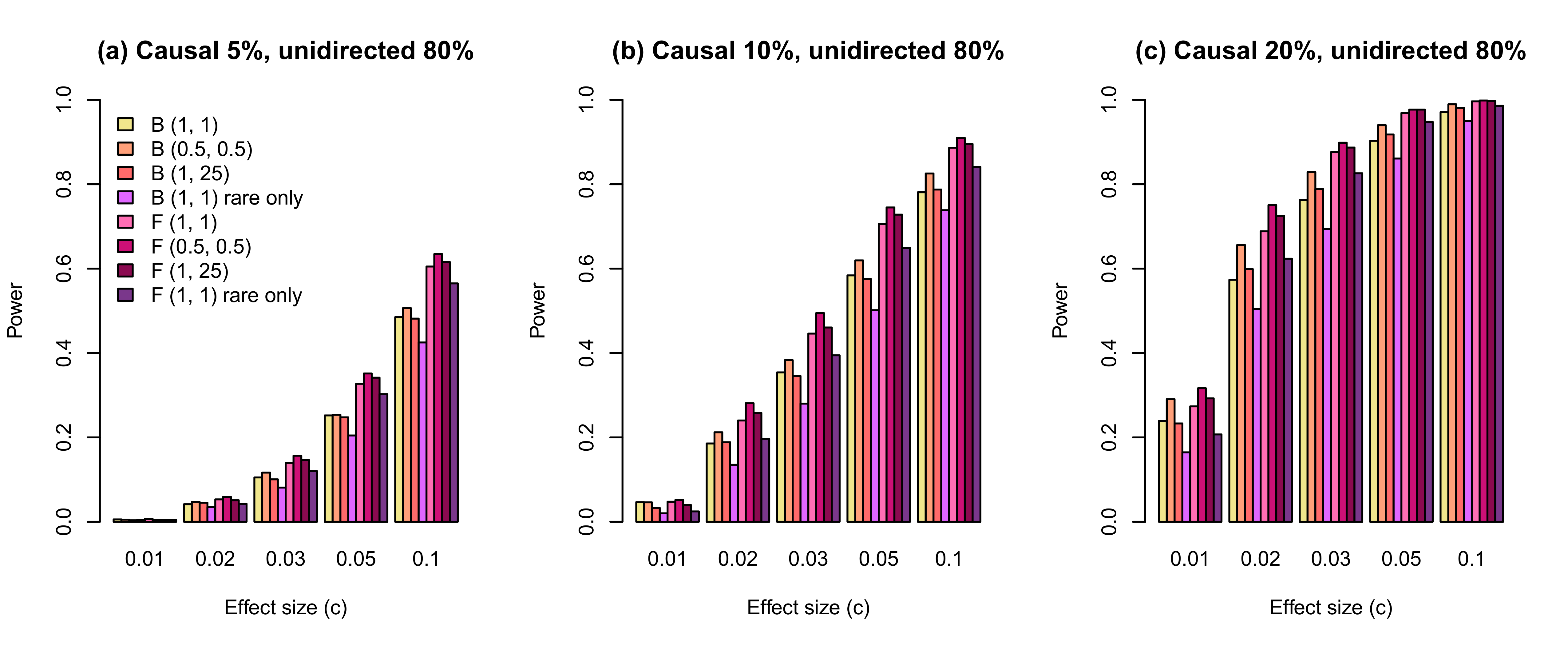

Supplement: S2 Fig — Proportion of causal variants is the proportion of all variants within the region (all variants = 100%). Other model parameters and notations are as in Fig 2. (TIF) [file pone.0190486.s002.tif]

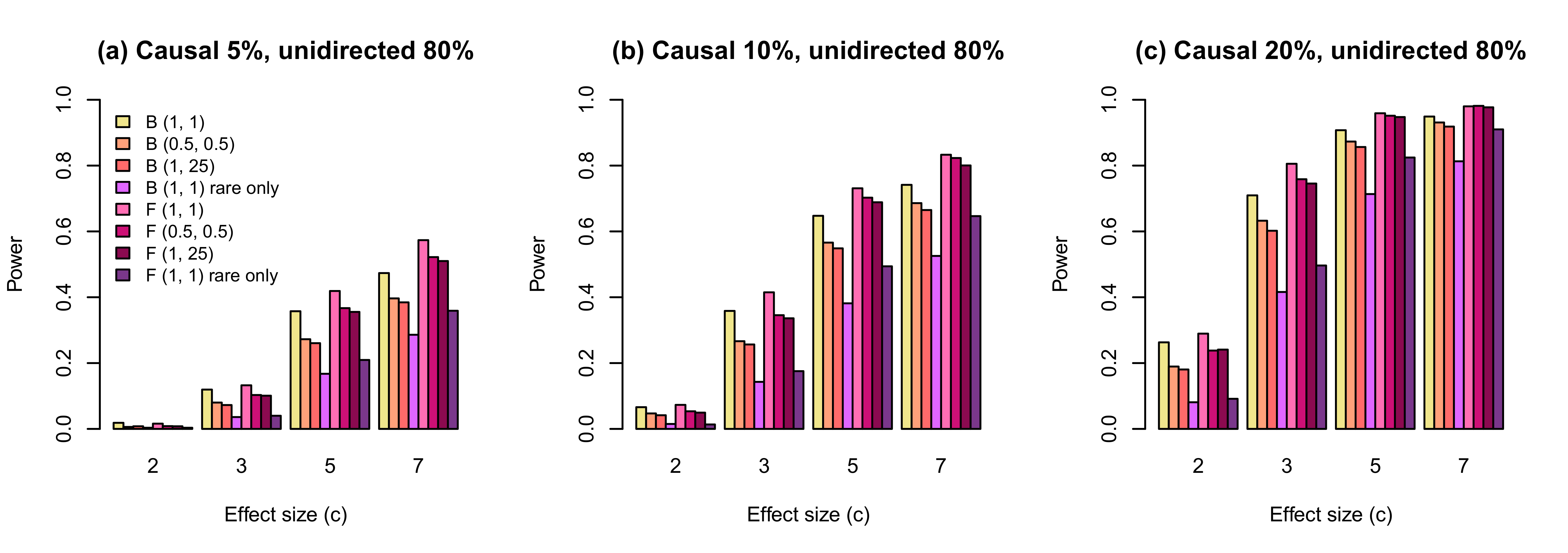

Supplement: S3 Fig — Proportion of causal variants is the proportion of all variants within the region (all variants = 100%). Other model parameters and notations are as in Fig 2. (TIF) [file pone.0190486.s003.tif]

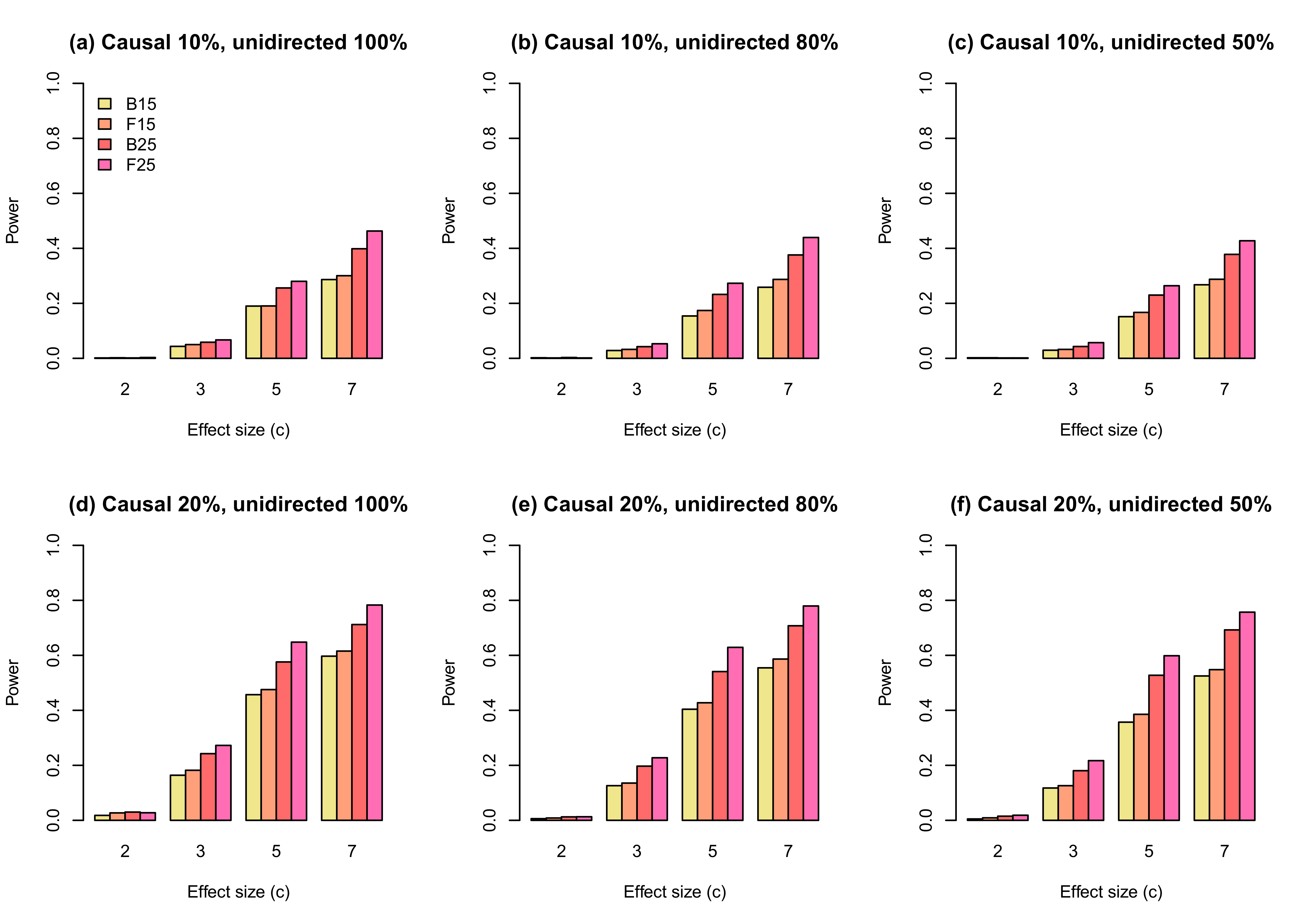

Supplement: S4 Fig — Unweighted FLM was used on familial data. B: B-spline basis functions; F: Fourier basis functions. The effect size for the j-th variant was modeled as |βj| = log(s)|log10(MAFj)|/2 using rare variants. Other model parameters and notations are as in Fig 2. (TIF) [file pone.0190486.s004.tif]

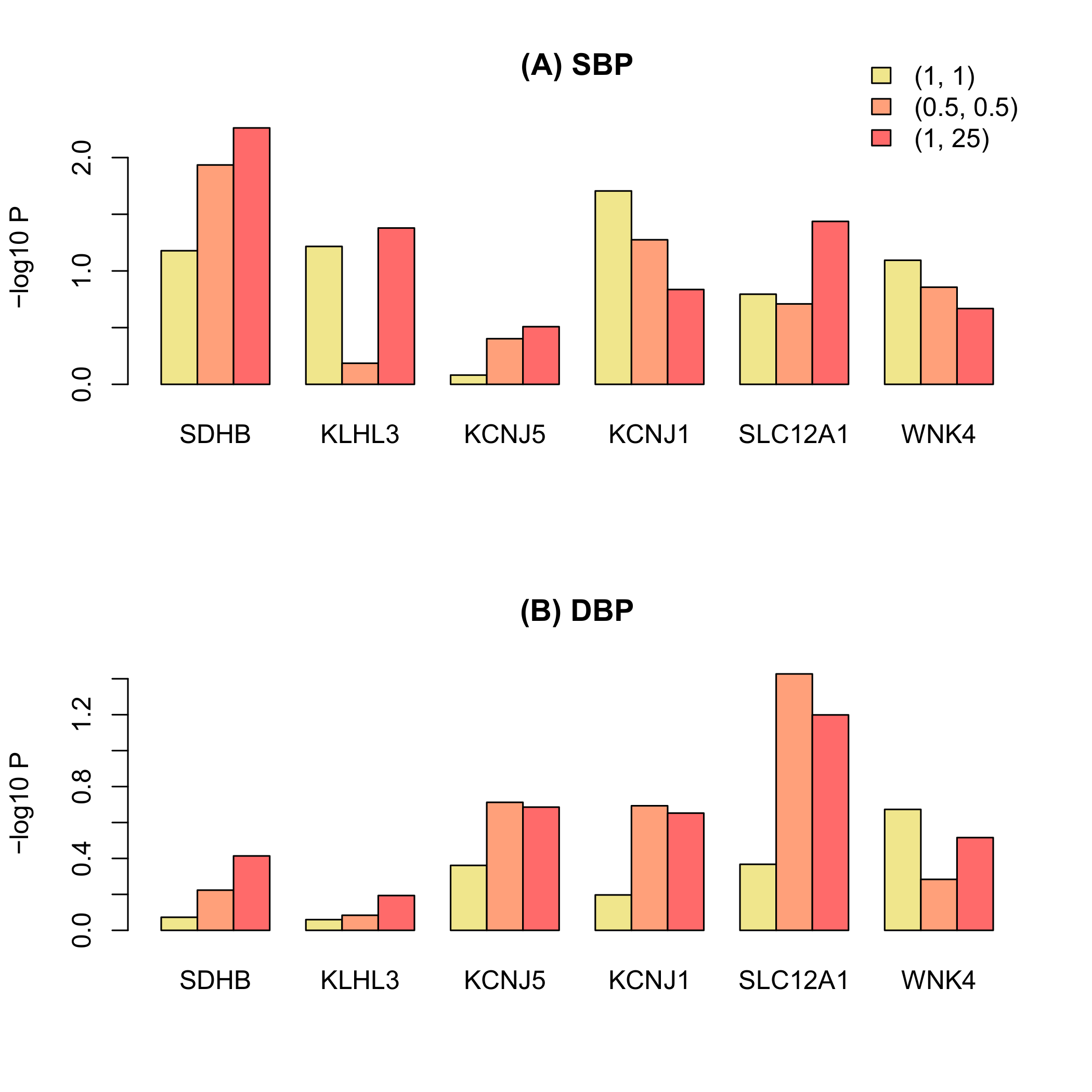

Supplement: S5 Fig — The differently weighted FLM based on the B-spline basis functions was used. The notations of the models are the same as in Fig 2. (TIF) [file pone.0190486.s005.tif]
